# The use of feedback in teaching undergraduate dental students: feedback sandwich or Ask-Tell-Ask model?

**DOI:** 10.1186/s12903-023-03141-5

**Published:** 2023-06-23

**Authors:** Galvin Sim Siang Lin, Wen Wu Tan, Hasnah Hashim, Chan Choong Foong

**Affiliations:** 1 Department of Dental Materials, Faculty of Dentistry, Asian Institute of Medicine, Science and Technology (AIMST) University, 08100 Bedong, Kedah Malaysia; 2 Department of Dental Public Health, Faculty of Dentistry, Asian Institute of Medicine, Science and Technology (AIMST) University, 08100 Bedong, Kedah Malaysia; 3 Medical Education and Research Development Unit (MERDU), Faculty of Medicine, Universiti Malaya, Kuala Lumpur, 50603 Malaysia

**Keywords:** Dental education, Dental materials, Feedback, Teaching method, Undergraduate

## Abstract

**Background:**

Feedback is regarded as a key component of formative assessment and one of the elements with the greatest impact on students’ academic learning. The present study aimed to evaluate and compare students’ perceptions of the use of two feedback models, namely feedback sandwich and Ask-Tell-Ask (ATA), in teaching dental materials science courses.

**Methods:**

All undergraduate second-year dental students were invited to participate in the dental materials science practical session and were randomly allocated into two groups: Group 1 (feedback) sandwich and Group 2 (ATA). The session began with the teacher giving a short briefing on the commonly used dental materials, followed by a short demonstration of the manipulation of those materials. Students were then allowed to mix and manipulate the materials, and teachers provided feedback accordingly. At the end of the session, 16 close-ended (five-point Likert scales) and an open-ended questionnaire were distributed to students to evaluate their perceptions of the feedback given. Internal reliability of the questionnaire items was evaluated using Cronbach’s alpha. Mean feedback scores were analysed using an independent t-test with ANCOVA for controlling gender and ethnicity. Thematic analysis was used to code the qualitative data.

**Results:**

Sixty-nine students participated in the present study with the majority being females (72.5%) and Chinese (79.7%). Cronbach’s alpha analysis suggested removing three Likert-scale items, with the remaining 13 items being accepted. Generally, no significant difference was noted between the two groups (*p* = 0.197), but three items were found to be significant (*p* < 0.05), with higher mean scores in the feedback sandwich group. Moreover, no significant difference was noted between the two feedback models (*p* = 0.325) when controlling gender and ethnicity. The open-ended question showed that students in the feedback sandwich group expressed greater positive perceptions.

**Conclusion:**

Although students generally had positive perceptions of both feedback models, they tended to favour the feedback sandwich. Neither gender nor ethnicity affected the students’ perceptions of the two feedback models.

## Background

Dental education has evolved from traditional pedagogy with major didactic information acquisition towards the accomplishment of learning outcomes in competency-based education [1]. Dental students are expected to acquire certain competencies prior to graduation, and dental schools are responsible to ensure that future dental graduates are qualified and competent to practise independently in a safe manner and committed to continuing professional development [1, 2]. Therefore, a rigorous and comprehensive assessment system focusing on the use of assessments for learning is required for such an educational system. It is also crucial to pay more attention to formative assessments rather than solely evaluating students based on summative assessments since formative assessments may help steer students in attaining the essential competencies [3]. One way to offer effective formative assessment is through timely, precise, and focused feedback [4, 5].

Undeniably, feedback is regarded as a key component of formative assessment and one of the elements with the greatest impact on learning [6]. Feedback is a systematic method for evaluating performances in relation to intended learning outcomes. To enhance students’ learning, high-quality formative feedback is designed to assist them to recall their previous actions and reflect on their current performance [7]. Moreover, feedback is effective in assisting and directing students towards more significant self-directed assessment-seeking behaviour, which is essential in competency-based education [5]. Without detailed and helpful feedback to students, continuous performance enhancement may not be achievable. Nevertheless, both teachers and students must possess the necessary skills to provide and receive feedback to ensure effective learning [8]. Research has shown that students’ academic performance is greatly improved through feedback. For instance, a previous study found that feedback enhanced the operative dentistry performance of preclinical dental students [9]. Moreover, another study found that dental students highly valued the feedback they received and linked it to increased individual performance [10].

Feedback may be delivered in a variety of ways which include the feedback sandwich model and the Ask-Tell-Ask (ATA) model. The term "feedback sandwich" appears to have originated in the 1940s [11], but it was not until Mary Kay Ash, the founder of Mary Kay Cosmetics, popularized it in her book "Mary Kay on People Management" [12], where she emphasised the significance of sandwiching criticism between compliments. Schwenk and Whitman then highlighted the feedback technique as a teaching strategy to enhance medical teaching in 1987 [13]. The feedback sandwich model entails beginning with praise, offering criticism, and then concluding with positive comments [14]. It has been proposed that starting and finishing feedback with praise enhances students' comfort and trust, increases their tolerance to criticism, reduces the potential harm that criticism can do to their self-esteem, and boosts their learning motivation [14, 15]. In addition, a previous study conducted among undergraduate healthcare students revealed that students preferred feedback beginning with a positive statement to capture their attention, followed by recommendations for improvement [8].

On the other hand, the ATA feedback was designed as a notion for bidirectional feedback that facilitates feedback conversation [7]. It was first implemented at the Cleveland Clinic in 2005 to assist teachers in enhancing medical students' reflective and self-evaluation abilities [4]. The three-step ATA feedback system is a learner-centred strategy for altering and reinforcing the behaviour. In the first step, the teacher would request the student to evaluate his or her performance, followed by the teacher acknowledging and giving comments on the student’s performance based on the teacher’s observations. Subsequently, the teacher probes the student's comprehension and encourages them to develop a strategy for improvement [16].

Dental materials science is a crucial preclinical course in most undergraduate dental programmes that integrates the concept of chemical engineering and materials science into the field of dentistry [17]. Future dental graduates should be competent in selecting, mixing, and manipulating commonly used dental materials to provide better oral healthcare services to the public. It is reasonable to assert that understanding the manipulation and clinical application of dental materials enhances clinical competency [18]. One may hypothesise that feedback given during teaching dental materials science courses would possibly enhance students’ competencies in mixing and manipulating dental materials. Nonetheless, it is still scarce in the literature where the strength of the evidence rests and what the most effective feedback for the teaching of dental materials science courses among undergraduate dental students would be.

Nevertheless, it is critical to consider individual differences that may affect how feedback is received and perceived when evaluating students’ perceptions of various feedback models. Ethnicity is an important factor, as a previous study showed that cultural values and norms can influence attitudes towards feedback. According to the study, Asian medical students typically respond better to feedback that is given in a respectful and positive manner [19]. Gender may also play a role in feedback preferences, with male students generally preferring elicitation feedback over female students [20]. Thus, the present study aimed to evaluate and compare students’ perceptions of the use of two feedback models, feedback sandwich and ATA feedback, in teaching dental materials science courses, while controlling for the possible effects of gender and ethnicity.

## Methods

### Sampling and participants

The present study was conducted among Year 2 Bachelor of Dental Surgery (BDS) students at the Faculty of Dentistry, Asian Institute of Medicine, Science and Technology (AIMST) University, Malaysia, with the ethical approval code: AUHEC/FOD/2023/17. All 70 students were invited to participate in the dental materials science practical session. To avoid potential bias in group distribution, each student was randomly allocated into two different groups using a computer-generated random number: Group 1 – feedback sandwich and Group 2 – ATA.

### Design and setting

The dental material science practical session took place in the faculty simulation laboratory. Students were divided into three batches and each session lasted around two hours. The session began with the teacher giving a short briefing using a PowerPoint slide presentation on commonly used dental materials in clinical settings. This is followed by a short manual demonstration on the manipulation of the materials performed by the teacher. The session included mixing and manipulating dental composite resin, glass ionomer cement, zinc oxide eugenol cement, calcium hydroxide cement, alginate impression material and zinc oxide eugenol impression paste. The content of the practical session was discussed and validated among the faculty members of the department to ensure that the learning activities were in line with the course learning outcomes, which was further approved by the department head. Next, students in the feedback sandwich and ATA groups were given the opportunity to mix and manipulate the different materials by themselves at their respective stations. A total of six stations were set up for them to manipulate the six different types of materials and the students were given 10 min at each station. During the session, teachers closely monitored each student’s performance at the respective station and provided feedback accordingly. This included evaluating the students’ skills in mixing the materials step-by-step and manipulating the materials within the manufacturers’ recommended setting time.

Prior to the session, two teachers (feedback providers), who were also faculty lecturers with post-graduate dental qualifications, read, familiarised, and practised by themselves with characteristics of effective feedback in the dental education context. They discussed various interpretations to align themselves with the timing and language style while delivering the feedback. For the feedback sandwich group, the first teacher adopted feedback sandwich model proposed by Schwenk and Whitman in 1987 [13], where positive feedback was given by the teacher to students based on their performances on the manipulation of each type of material before and after the negative or constructive feedback. Thus, positive feedback serves as a cushion for negative ones. Meanwhile, the ATA feedback model was employed by another teacher who first asked the student to self-assess his or her performance on the manipulation of different types of materials, followed by feedback from the teacher and reinforcement on the steps that the student did well. Finally, the teacher asked the student to develop a plan to improve their future performance.

### Questionnaire design

The questionnaire used in the present study was developed and modified from previous similar studies [21, 22]. It consisted of 16 close-ended questions and an open-ended question to evaluate students’ perceptions of the two different feedback models. Each close-ended question had five responses using a five-point Likert scale, ranging from strongly agree to strongly disagree. The scores distribution was strongly agree: 2, agree: 1, neutral: 0, disagree: -1, and strongly disagree: -2. The open-ended question was: What do you think work well in the feedback given? Content validation of the questionnaire was performed by two experts (faculty members) and the internal reliability of the questionnaire items was evaluated using Cronbach’s alpha. A coefficient value of > 0.7 was deemed acceptable reliability.

### Data collection & analysis

At the end of the session, the questionnaire was distributed using an online Google Form after obtaining informed consent. The collected data were analysed using IBM Statistical Package for the Social Sciences (SPSS) for Windows, Version 29.0. (Armonk, NY: IBM Corp., USA). The mean feedback scores were analysed using an independent t-test. Furthermore, an adjusted mean using ANCOVA for controlling gender and ethnicity was employed, and thematic analysis was used to code the qualitative data.

## Results

A total of 69 students participated in the present study with a respondent rate of 98.6%. Among the 69 students, 50 being females (72.5%) and 19 being males (27.5%). The ethnicity of the students was predominantly Chinese, accounting for 55 (79.7%) students. The study consisted of two intervention groups: the feedback sandwich group (34 students, 49.3%) and the ATA group (35 students, 50.7%), respectively. Cronbach’s alpha analysis suggested removing three Likert-scale items: “*The feedback given makes me feel discomfort during the session*”, “*The feedback given makes me feel threatened to improve my knowledge and skills in dental materials*”, and “*I feel uncomfortable when teachers provide direct feedback without praise*”, with the remaining 13 items being accepted. Hence, the maximum and minimum scores were 26 and – 26.

Responses from all the 13 items were scored and summed. The difference in perception scores between the feedback sandwich group (mean score = 24.7) and the ATA group (mean score = 23.2) was not statistically significant (*p* = 0.197) as listed in Table 1. Moreover, the result remained unchanged even after controlling for the effects of gender and ethnicity (*p* = 0.325). However, based on the individual item comparison in Table 2, significant differences between feedback sandwich and ATA groups were noted, particularly for item 2 "*The feedback given allows me to improve my performance in mixing and manipulating dental materials*" (*p* = 0.036), item 5 "*The feedback given motivates me to improve my technique in mixing and manipulating dental materials*" (*p* = 0.024), and item 12 "*I enjoyed the way the teacher provides feedback during the session*"(*p* = 0.019). Notably, the mean scores for each of these three items were higher in the feedback sandwich group than in the ATA group, suggesting more favourable perceptions in the feedback sandwich model than the ATA model.

**Table 1:** Comparison of the mean feedback scores between Ask-Tell-Ask and feedback sandwich Models with controlling for potential confounders

Model	*n*	Mean (SD)	Mean diff. (95% CI)	*t* stat (*df*)	*p-*value^♦^	Adjusted mean^♯^ (95% CI)	Adjusted mean diff. (95% CI)^╪^	*F* stat^♯^ (*df*)	*p-*value^♯^
Ask-Tell-Ask	35	23.2 (5.17)	-1.4 (-3.7, 0.7)	-1.3 (67)	0.197	19.3 (16.0, 22.5)	-1.1 (-3.2, 1.1)	0.98 (1,64)	0.325
Feedback Sandwich	34	24.7 (3.98)				20.3 (16.8, 23.8)			

^♦^Independent t-test with equal variances assumed

^♯^Adjusted mean using ANCOVA controlling for gender and ethnicity

^╪^Bonferroni adjustment for 95% confidence interval for difference

**Table 2:** Differences in perception on feedback sandwich and Ask-Tell-Ask models

Questionnaire Items	Feedback Sandwich mean (s.d.)	Ask-Tell-Ask mean (s.d.)	*p-*value^○^
1. The feedback given allows me to improve my understanding of the technique in mixing and manipulating dental materials	1.91 (0.288)	1.74 (0.443)	0.065
2. The feedback given allows me to improve my performance in mixing and manipulating dental materials	1.91 (0.288)	1.71 (0.458)	0.036^*^
3. The feedback given allows me to reflect on my technique in mixing and manipulating dental materials	1.85 (0.359)	1.80 (0.406)	0.569
4. The feedback given encourages me to learn more about dental materials science	1.76 (0.496)	1.77 (0.426)	0.952
5. The feedback given motivates me to improve my technique in mixing and manipulating dental materials	1.94 (0.239)	1.74 (0.443)	0.024^*^
6. The feedback given allows me to identify my mistakes in mixing and manipulating dental materials	1.88 (0.327)	1.74 (0.443)	0.141
7. The feedback given makes me feel enjoyed during the session	1.85 (0.359)	1.69 (0.471)	0.102
8. The feedback given allows me to be well-prepared into clinical setting	1.62 (0.493)	1.66 (0.591)	0.764
9. The feedback given allows me to think beyond what is being taught in the classroom	1.59 (0.557)	1.60 (0.497)	0.926
10. I am able to pay close attention to the feedback given during the session	1.82 (0.387)	1.74 (0.443)	0.424
11. I prefer the teacher to provide positive feedback and praise during the session	1.24 (1.017)	1.03 (0.857)	0.364
12. I enjoyed the way the teacher provides feedback during the session	1.85 (0.359)	1.60 (0.497)	0.018^*^
13. The feedback provided by the teacher is uncleared	1.59 (0.783)	1.60 (0.497)	0.941

*s.d.* standard deviation

^○^Independent t-test with equal variances assumed

^*^Significance at 0.05

Although students in both feedback groups generally had favourable perceptions of the practical session, it can be seen from the open-ended question that students in the feedback sandwich group expressed greater positive perceptions. In the feedback sandwich group, students enjoyed the session and felt more confident when positive feedback was provided before and after the negative feedback. Some of the quotes are as follow:

“The feedback given by the lecturer was useful. The doctor (teacher) praised us first and then pinpointed our mistakes. It really boosted our confidence as a year 2 student who is practicing the mixing of the restoration material, as a first timer.”

“The feedback that Dr gave was very clear and motivating, I think a teaching way like this let me feel really enjoy and comfortable.”

On the other hand, students in the ATA group appreciated the explanation and feedback given by the teacher based on their performance. Some of the quotes are as follow:

“Teacher gave good feedback and comments.”

“Dr’s explanation was clear, and everyone had the chance to do well…”

## Discussion

The present study is the first of its kind to compare the perceptions of undergraduate dental students towards two different feedback models during a dental materials science practical session, namely the feedback sandwich and ATA. Undeniably, providing useful and effective feedback is crucial to dental education as it can encourage active learning and promote the accomplishment of specific learning outcomes [7]. Effective feedback can assist students in reflecting on their learning experience and identifying areas where there is a gap between anticipated learning outcomes and the actual outcomes [5]. It is important to emphasise feedback in dental education as effective feedback perpetually promotes good clinical practice in dental settings [23]. A learning cycle that lacks feedback leads to stunted knowledge growth among learners, which may later have detrimental effects on overall patient care [24].

Although generally both feedback models received positive perceptions with no significant difference among undergraduate students, three questionnaire items were found to be significantly different between feedback sandwich and ATA models based on the individual questionnaire item comparison. The majority of the students in the feedback sandwich group (mean score = 1.91) agreed that the feedback given allows them to improve their performance in mixing and manipulating dental materials as compared to students in the ATA group (mean score = 1.71). Such a finding is in accordance with a previous study conducted among third-year medical students who agreed that feedback sandwich would help to improve their performance on clinical note-writing [15]. Similarly, another study comparing the feedback sandwich and learning conversation structured methods in competency-based basic life support training for healthcare students was carried out in the United Kingdom [25]. The study showed that all healthcare students who received feedback using the feedback sandwich model concurred that it had improved their ability to perform basic life support.

Moreover, a significant number of students in the feedback sandwich group (mean score = 1.94) agreed that the feedback provided motivated them to improve their technique in mixing and manipulating dental materials as compared to students in the ATA group (mean score = 1.74). This can be explained by self-determination theory, acknowledging the importance of motivation as a mean to achieve learning goals [26]. The development of intrinsic motivation is thought to benefit greatly from effective feedback [27]. Intrinsic motivation is the tendency to perform an action out of pleasure or delight, for its own sake, or without receiving any kind of external rewards or incentives [28]. It is believed that intrinsically motivated students perform better in their academic learning. In fact, several studies have evaluated the effects of positive feedback on students’ motivation and revealed that positive feedback improved their motivation as it reinforces a sense of competence among students [29, 30]. Indeed, beginning and ending with praise while giving feedback builds trust and comfort in students’ learning processes, increases their acceptability of negative feedback, and motivates them to participate in the learning process [31–33]. However, when using the feedback sandwich model, one should be cautious because positive comments can dilute negative ones, causing corrective comments to be overlooked.

Surprisingly, most of the students in the feedback sandwich group (mean score = 1.85) agreed that they enjoyed the way the teacher provided feedback during the session as compared to students in the ATA group (mean score = 1.60). This is also supported by the open-ended responses whereby students in the feedback sandwich group enjoyed the session and felt more confident when positive feedback was given before and after negative feedback. Scholars have argued that adopting the ATA model for feedback improves students' accountability for their learning since they must begin the feedback dialogue with their own self-evaluation [16, 34]. The combination of self-evaluation and external feedback from teachers will help students hone their self-evaluation skills. This method is also claimed to be more student-centred which facilitates self-reflection and skill evaluation [4]. However, such an argument contradicts the present findings as students in the current study seemed to enjoy ‘sugar-coated’ positive feedback. Thus, the authors hypothesized that Asian students prefer praise and positive feedback in learning over a learner-centred feedback approach. Previous research indicated that Asian students are more praise and token oriented [35, 36]. Another plausible explanation could also be the fact that feedback sandwich model was found to be easier for teachers to understand and practice [25]. It is reasonable to argue that a teacher would be able to effectively implement the specific feedback model among students if they have a solid understanding of it. Nevertheless, such an argument must be confirmed or refuted by comparing these two feedback models when teaching more sophisticated practical sessions.

In addition, no significant difference was noted when comparing the total perception scores obtained from the two feedback models while controlling potential confounding effects of gender and ethnicity. This finding defies previous research highlighting that male and female students had distinct interests and learning styles. Male students tend to be more engaged in their learning and interact with teachers more than their female counterparts [37]. Male students were also found to have the propensity to pay more attention to their environment and external factors, whereas females are more prone to shift their attention inward and reflect their thoughts, feelings, and behaviour [38]. Furthermore, ethnic variations may affect students’ learning preferences. For instance, a small-scale study among Malaysian students indicated that Indian students place greater value on corrective feedback in oral communication compared to Malay or Chinese students [39]. Despite that, the present study did not identify any significant effects of gender and ethnicity on total feedback scores at the univariable level. Nonetheless, it is important to acknowledge that a disproportionate distribution of participants across these categories may have influenced the present results. With a smaller number of male students than females, such an imbalance could contribute to the lack of significance observed in the present study. Furthermore, most students were Chinese, which reduced ethnic diversity and could have influenced the lack of substantial impacts. The present study analysed these potential confounders in a multivariable analysis to obtain adjusted mean scores for both feedback techniques, providing a more accurate representation of differences in perceptions than crude mean scores.

In general, both feedback models have several advantages and disadvantages. The feedback sandwich model's proponents contend that beginning and concluding with specific praise builds the receiver's comfort and trust, increases their openness to criticism and reduces the potential harm that criticism might do to their self-esteem [15]. Discouraging criticism that undermines self-esteem lowers learning and performance [40], especially in young students (preclinical year students, for instance) who may not be emotionally mature. In contrast, opponents of the feedback sandwich model asserted that it obscures the essential point of the feedback since students receive more positive feedback than negative, leading them to overlook the corrective comments [15, 40]. Moreover, such a feedback model does not provide recommendations for improvement or specific details of the learner's strengths. [40]. On the other hand, the ATA model requires students to initiate the feedback conversation with a self-reflection to evaluate their performance and identify knowledge gaps before receiving feedback from teachers. Students are more receptive to corrective feedback when they have self-identified areas for improvement, and assessment becomes a shared responsibility between teachers and students [4]. Students' feedback-seeking behaviours frequently increase as they become more self-reflective [41]. However, caution should be exercised when using the ATA model, as students may be subpar on unguided self-evaluation [42].

Several flaws were noted in the present study. First, the students were recruited from a single institution, and thus, the findings could not be generalised to all Malaysian dental students. Future research should include students from different dental schools in the country as well as comparing with students from other nations. It is worth noting that dental degrees are offered as post-graduate programmes in some countries and students’ previous educational backgrounds, maturity and life experience may affect their perceptions and utility of various feedback models [43]. Second, teachers’ attitude and tone when providing feedback may unintentionally influence the students' perceptions. It is also wise to explore teachers’ perceptions of using these two feedback models in teaching dental materials science courses. Third, the extent to which the mixing and manipulating of dental materials skills are retained is obviously of great significance, but it is uncertain from the results whether the appropriate feedback model would have an influence on skill retention. Moreover, the present study was conducted among preclinical year dental students and the findings might be different if it was conducted among clinical year students. Future studies can be conducted to explore dental students' perceptions of the use of various feedback models in clinical settings. A recent systematic review stated that limited feedback models (10%) have undergone empirical evaluation, and little is known about comparing the effectiveness of different models on students’ academic performance [44]. In addition, the impact of various feedback models on student’s attitude towards infection control and professionalism in the clinical setting should be assessed whenever possible. Future studies should also consider longitudinal research to compare the effectiveness of different feedback models with additional models that may be considered such as the Pendleton and Feedforward models.

## Conclusion

Although students generally had positive perceptions towards both feedback models, it is apparent that students tended to favour the feedback sandwich model more. Moreover, neither gender nor ethnicity affected the students’ perceptions of the two feedback models. Nevertheless, future studies should be conducted on a larger population and compare different feedback models on students’ academic performances, as well as assess the views of teachers in using feedback in their instruction.

## Data Availability

All data generated or analysed during this study are included in this published article.

## Abbreviations

ATA: Ask-Tell-Ask
BDS: Bachelor of Dental Surgery
AIMST: Asian Institute of Medicine, Science and Technology University
SPSS: Statistical Package for the Social Sciences
